# Supplementary material for: Uncovering the Elusive Structures and Mechanisms of Prevalent Antidepressants
Source: Adv Ther (Weinh). Author manuscript; Available in PMC 2025 Feb 26. (PMC11864751; doi:10.1002/adtp.202400117)
Supplement: Supinfo [file NIHMS2011941-supplement-Supinfo.docx]

**Supplementary Information**

**Uncovering the Elusive Structures and Mechanisms of Prevalent Antidepressants**

Jieye Lin^1^, Guanhong Bu^1^, Johan Unge^1^ and Tamir Gonen^1,2,3^*

^1^ Department of Biological Chemistry, University of California, Los Angeles, 615 Charles E. Young Drive South, Los Angeles, California 90095, United States

^2^ Department of Physiology, University of California, Los Angeles, 615 Charles E. Young Drive South, Los Angeles, California 90095, United States

^3^ Howard Hughes Medical Institute, University of California, Los Angeles, Los Angeles, California 90095, United States

^*^ Corresponding Author T.G. tgonen@g.ucla.edu

**Supplementary Table S1** MicroED data statistics of five antidepressants.^a^

| Compound | **1** | **2** | **3** | **4** | **5** |
| --- | --- | --- | --- | --- | --- |
| Name | Reboxetine mesylate | Pipofezine dihydrochloride monohydrate | Ansofaxine hydrochloride dihydrate | Phenelzine sulfate | Bifemelane hydrochloride |
| Stoichiometric formula | C_20_H_27_NO_6_S | C_16_H_21_Cl_2_N_5_O_2_ | C_24_H_36_ClNO_5_ | C_8_H_14_N_2_O_4_S | C_18_H_24_ClNO |
| Mr | 409.50 | 388.30 | 454.03 | 234.28 | 305.80 |
| Temperature (K) | 80 | 80 | 80 | 80 | 80 |
| Crystal system | Monoclinic | Monoclinic | Monoclinic | Monoclinic | Orthorhombic |
| Space group | P 2_1_/c | P 2_1_/c | P 2_1_/c | P 2_1_/c | P bca |
| Unit cell lengths (Å) |  |  |  |  |  |
| a | 20.000(4) | 6.880(2) | 14.800(3) | 20.360(4) | 12.870(3) |
| b | 5.490(2) | 15.610(3) | 10.270(2) | 5.460(2) | 7.310(3) |
| c | 19.060(4) | 15.930(3) | 16.040(3) | 20.300(4) | 35.100(7) |
| Unit cell angles (°) |  |  |  |  |  |
| α | 90.00(3) | 90.00(3) | 90.00(3) | 90.00(3) | 90.00(3) |
| β | 107.34(3) | 97.22(3) | 95.31(3) | 111.21(3) | 90.00(3) |
| γ | 90.00(3) | 90.00(3) | 90.00(3) | 90.00(3) | 90.00(3) |
| Cell volume (Å^3^) | 1997.7(10) | 1697.3(7) | 2427.6(8) | 2103.8(10) | 3302.2(14) |
| No. of datasets merged | 7 | 4 | 3 | 1 | 3 |
| No. of observed reflections | 93185 | 32120 | 33547 | 21752 | 39392 |
| No. of unique reflections | 5384 | 3356 | 4068 | 7358 | 3423 |
| R_obs_ (%) | 27.7 | 33.6 | 28.8 | 17.2 | 28.2 |
| R_meas_ (%) | 28.6 | 35.5 | 30.8 | 21.2 | 29.6 |
| I/Sigma | 8.13 | 5.20 | 5.22 | 3.99 | 5.78 |
| CC_1/2_ | 99.4 | 97.8 | 98.1 | 98.9 | 98.9 |
| Completeness (%) | 99.8 | 99.7 | 86.3 | 83.2 | 97.7 |
| **Resolution (Å)** | **0.73** | **0.82** | **0.83** | **0.63** | **0.83** |
| **R_1_ (%)** | **19.3** | **15.9** | **18.7** | **20.2** | **19.6** |
| *w*R_2_ (%) | 52.1 | 40.2 | 41.1 | 48.7 | 47.1 |
| GooF | 2.134 | 1.439 | 1.422 | 1.423 | 1.712 |

**Notes:** MicroED structures **1** and **3**-**5** were solved by SHELXT,^1^ structure **2** was solved by SHELXD;^2^ All MicroED structures were refined by SHELXL.^3^

**Supplementary Table S2** Molecular Docking of five antidepressants and their target proteins

| **Ligand**^a^ | **Protein**^b^ | **Cofactor/ Coenzyme** | **Template PDB ID**^5-10^ **structures** | **Binding site^c^** |
| --- | --- | --- | --- | --- |
| **1R** | hNET | No | 4XNX | S1 |
| **1R** | hNET | No | 3GWV | S2 |
| **1S** | hNET | No | 4XNX | S1 |
| **1S** | hNET | No | 3GWV | S2 |
| **2** | hSERT | No | 5I73 | near S2 |
| **3R** | hSERT | No | 5I73 | S1 |
| **3R** | hNET | No | 4XNX | S1 |
| **3R** | hDAT | No | 4MMC | S1 |
| **3S** | hSERT | No | 5I73 | S1 |
| **3S** | hNET | No | 4XNX | S1 |
| **3S** | hDAT | No | 4MMC | S1 |
| **4*** | MAO-A | No | 2Z5X | C1 |
| **5** | MAO-A | Yes | 2Z5X | R1 |
| **5** | MAO-B | Yes | 1OJ9 | R1′ |

Notes: ^a^The ligand structures were directly extracted from MicroED structures of **1**-**3** and **5**, while the ligand structure of **4*** was extracted from PDB structure 2VRM (resolution: 2.30 Å). ^b^The protein structures were obtained from PDB database either by X-ray crystallography or AlphaFold, hNET (AF_AFP23975F1),^4^ hSERT (5I73),^7^ hDAT (AF_AFQ01959F1),^4^ MAO-A (2Z5X),^9^ MAO-B (1OJ9).^10^ ^c^The binding sites (docking center) was determined by aligning the protein with template structures, which was then used within an 18.75 Å × 18.75 Å × 18.75 Å grid box in AutoDock Vina 1.1.2.^11,12^


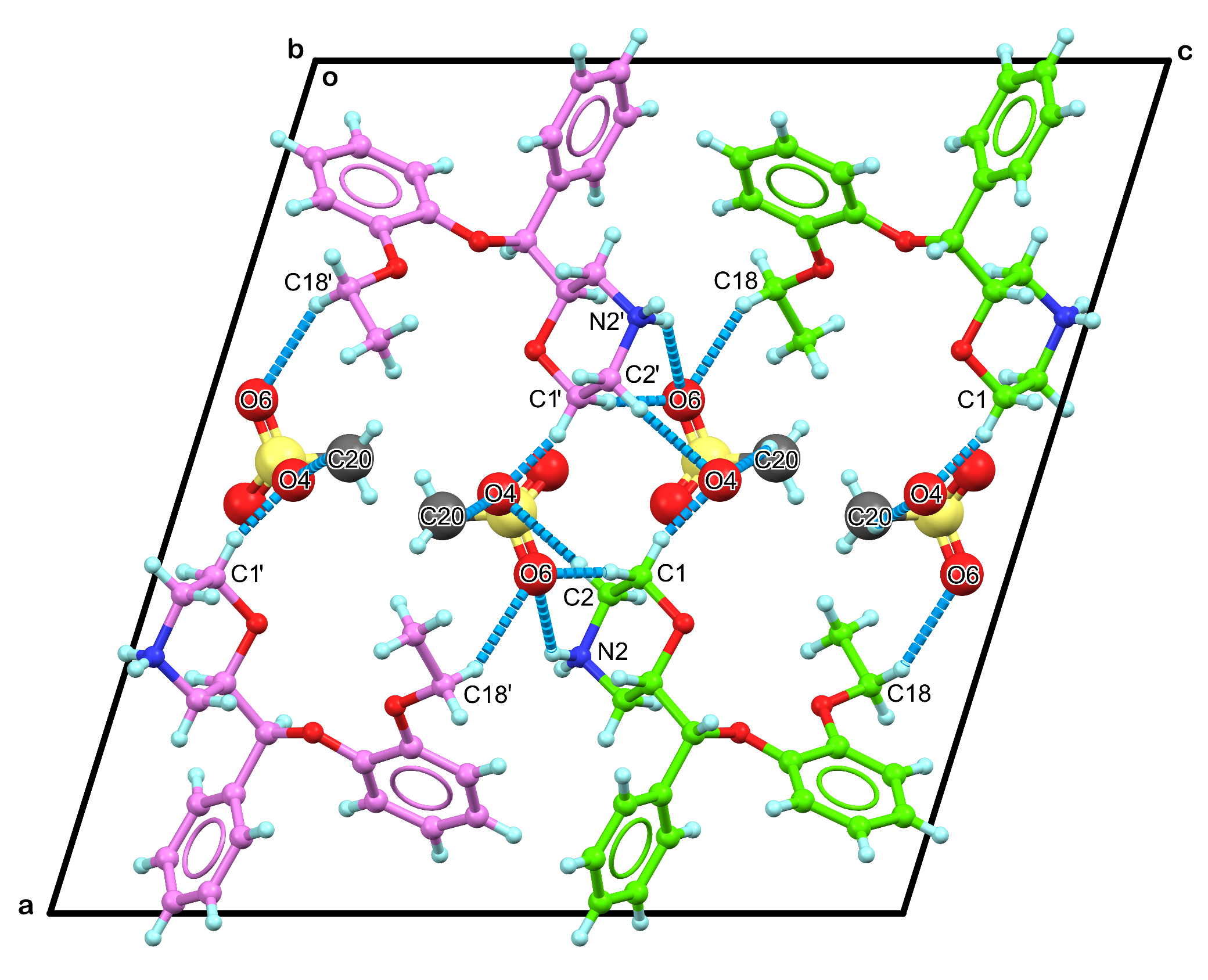


**Supplementary Figure S1** The packing diagram of **1**, viewed along the *b*-axis. Hydrogen bonding and selected dipole-dipole interactions were represented by the dashed lines in marine, **1R** was colored in green, **1S** was colored in violet.


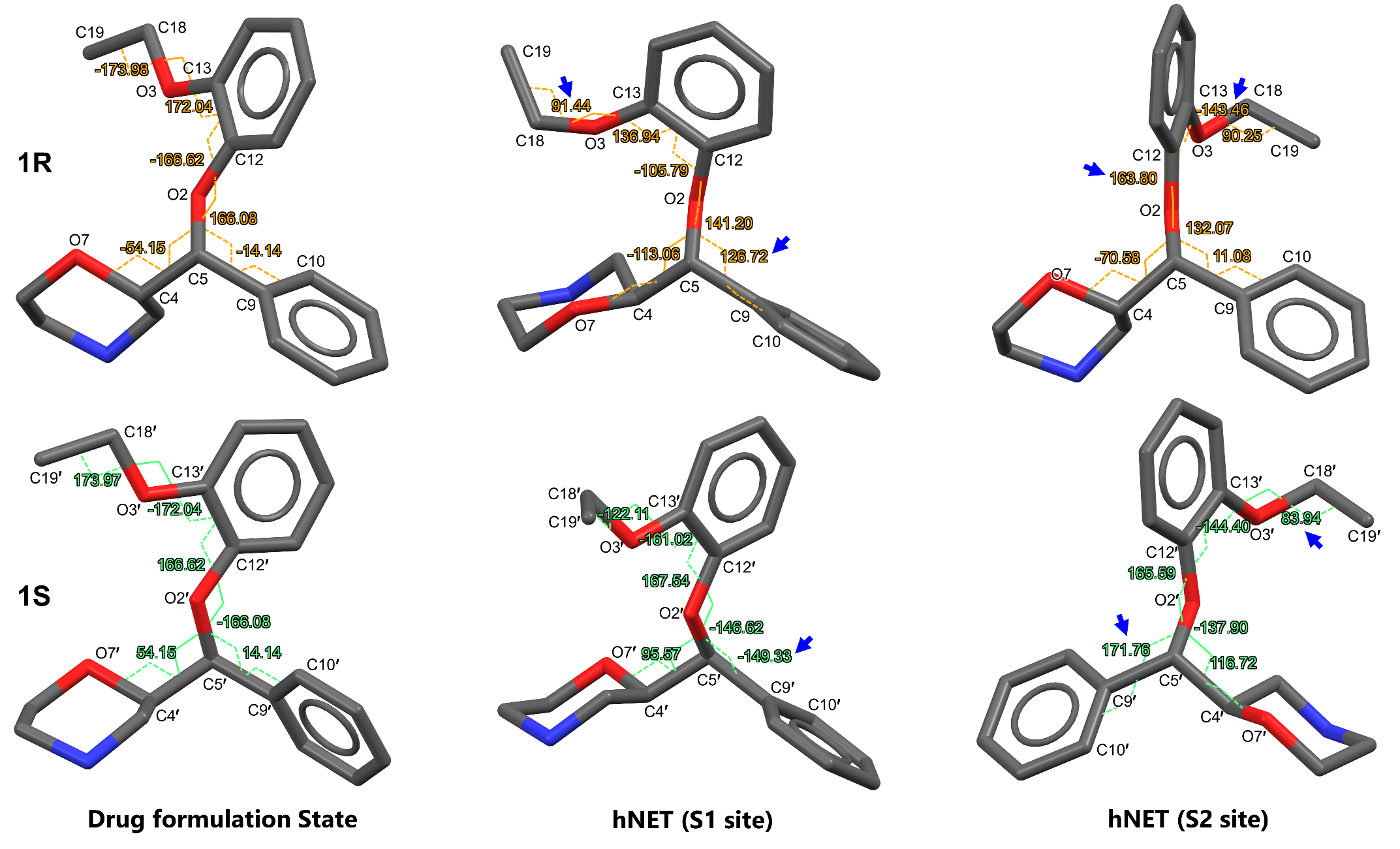


**Supplementary Figure S2** The major conformational changes between the crystal structures of **1** and their molecular docking structures in hNET. The major torsion changes were highlighted with blue arrows.


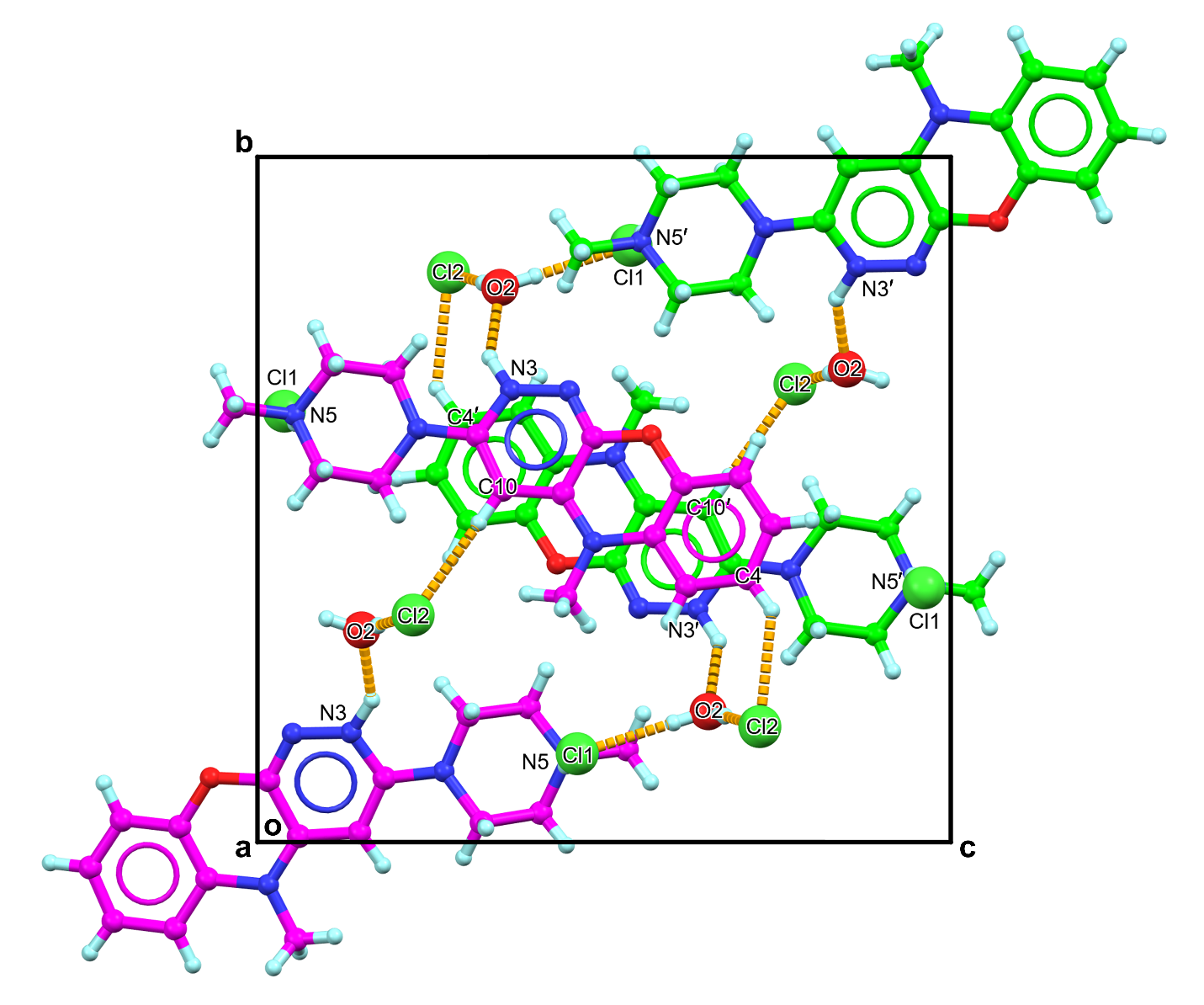


**Supplementary Figure S3** The packing diagram of **2**. Hydrogen bonding and selected dipole-dipole interactions were represented by the dashed lines in orange. **2a** was colored in magenta, **2b** was colored in green.


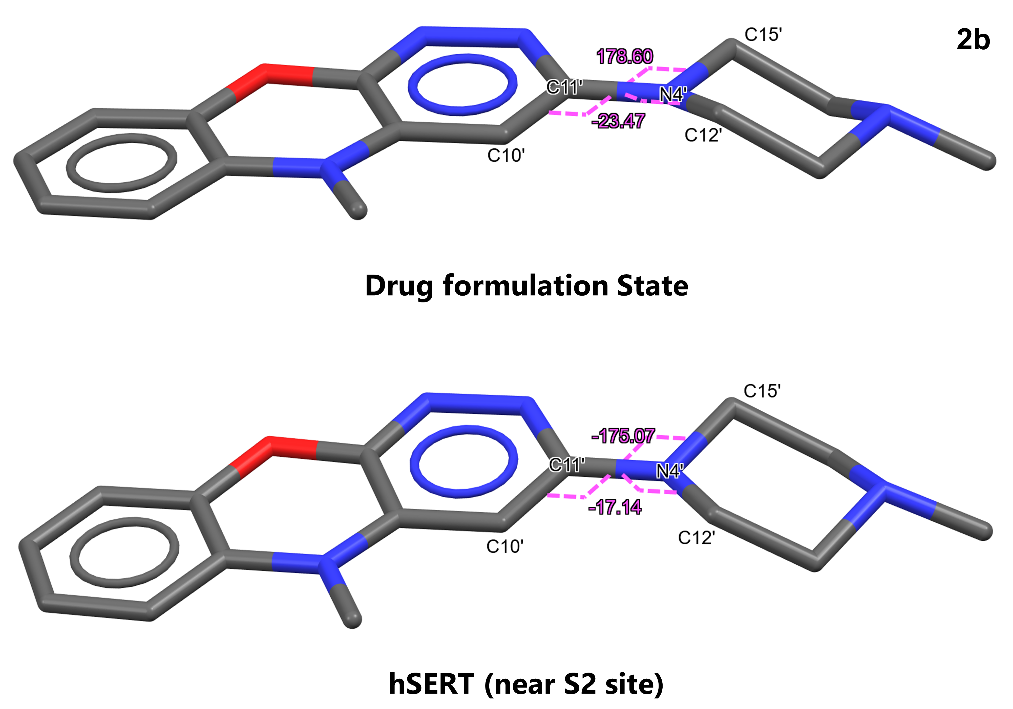


**Supplementary Figure S4** The conformational changes between the crystal structure of **2b** and its molecular docking structure in hSERT. The freely rotatable torsion angles (C10′‒C11′‒N4′‒C12′ and C10′‒C11′‒N4′‒C15′) were measured.


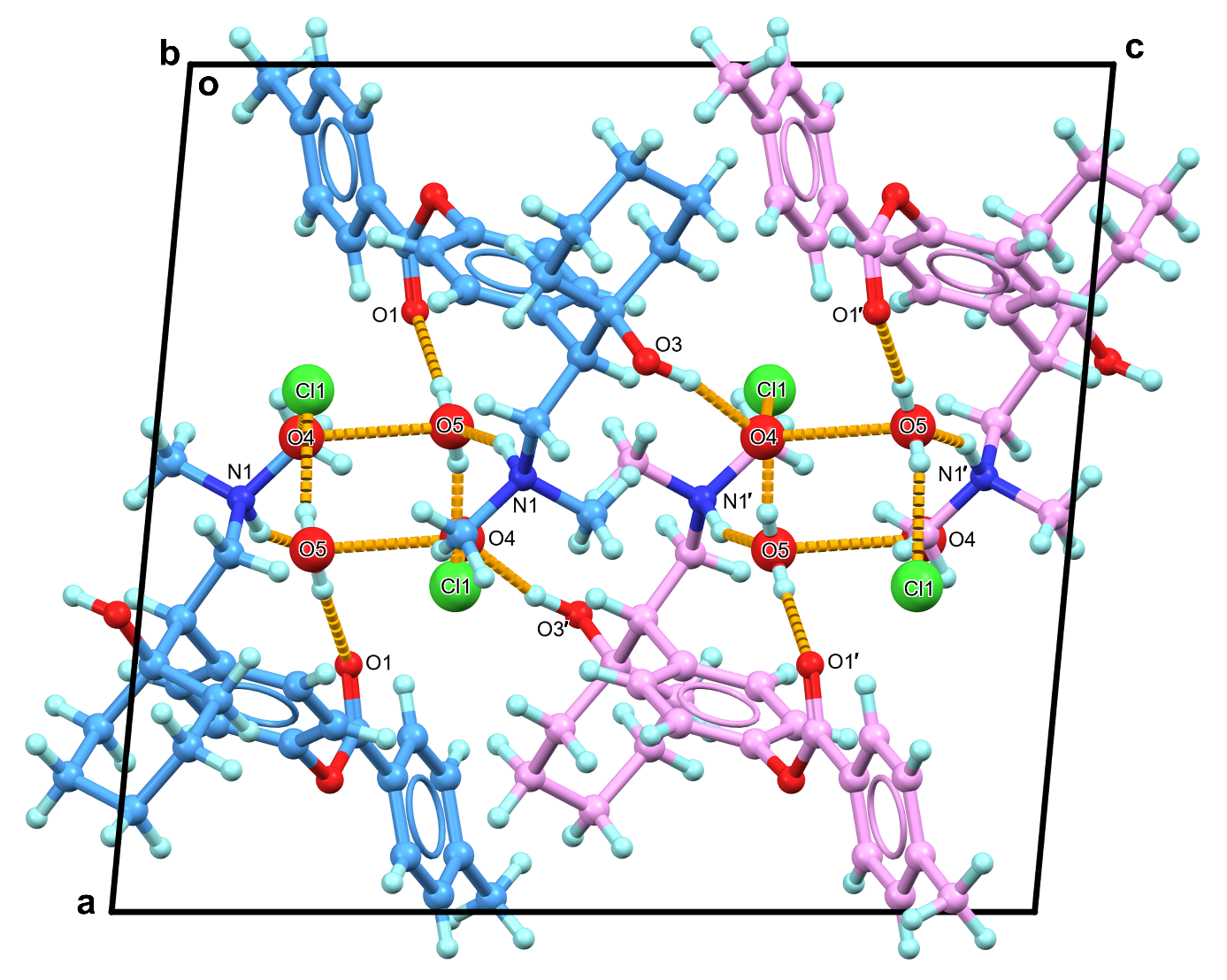


**Supplementary Figure S5** The packing diagram of **3**, viewed along the *b*-axis. Hydrogen bonding interactions were represented by the dashed lines in orange, **3R** was colored in blue, **3S** was colored in violet. The Cl1‒O4‒O5 is measured at 107.7° and O1‒O5‒Cl1 is measured at 112.8°.


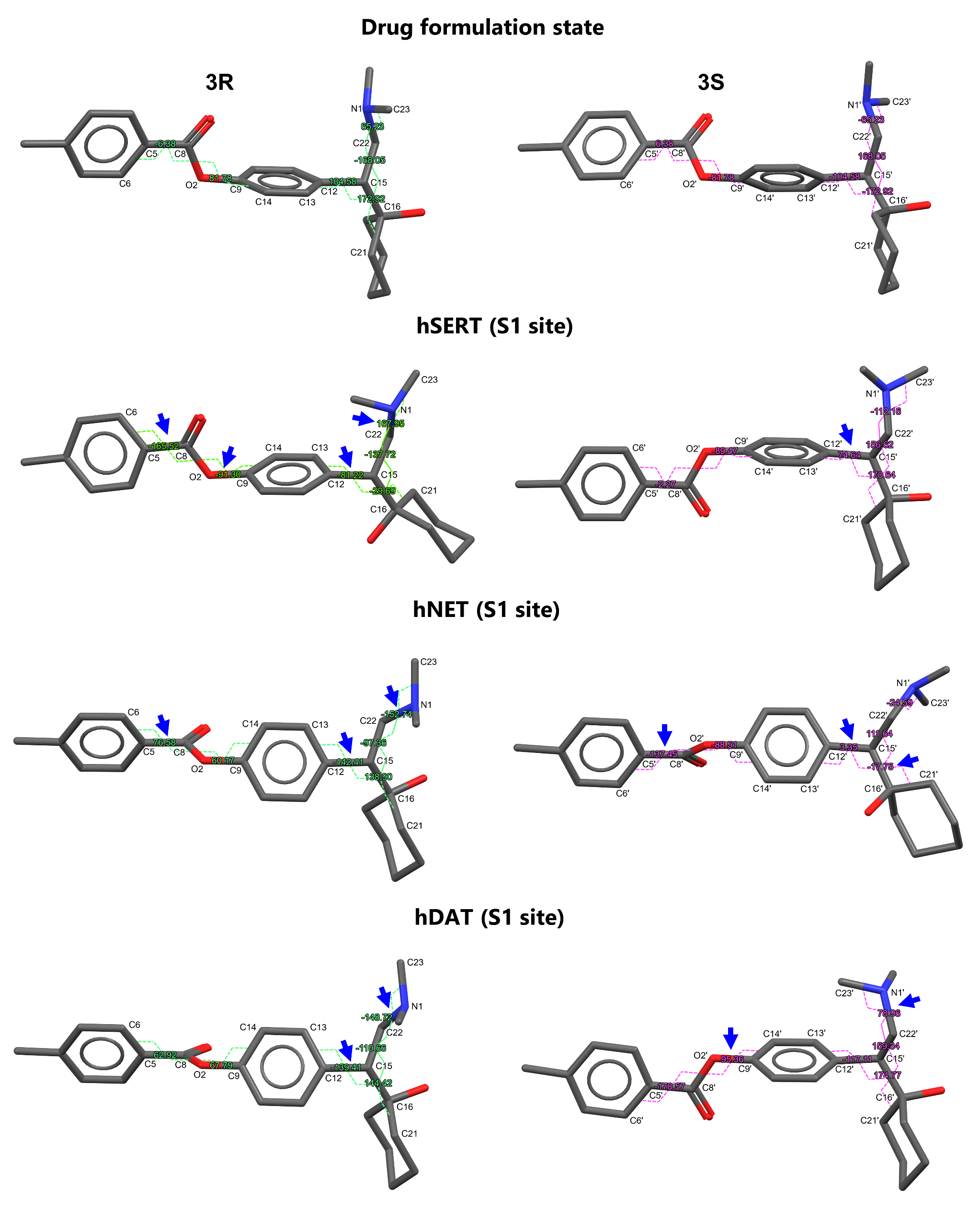


**Supplementary Figure S6** The major conformational changes between the crystal structures of **3** and their molecular docking structures in hSERT, hNET and hDAT. The major torsion changes were highlighted with blue arrows.


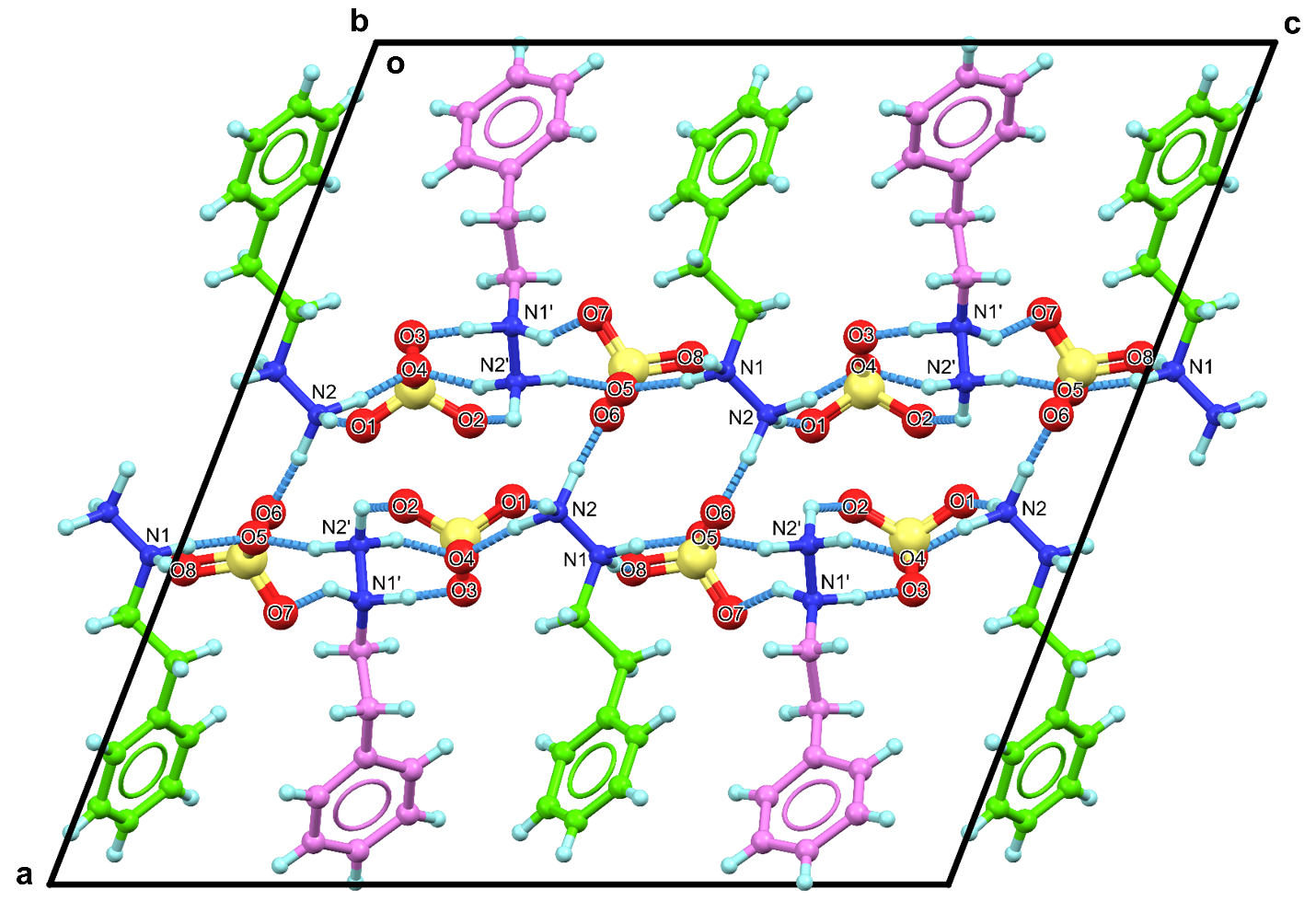


**Supplementary Figure S7** The packing diagram of **4**, viewed along the *b*-axis. **4a** was colored in green, **4b** was colored in violet. Hydrogen bonding interactions were represented by the dashed lines in marine.


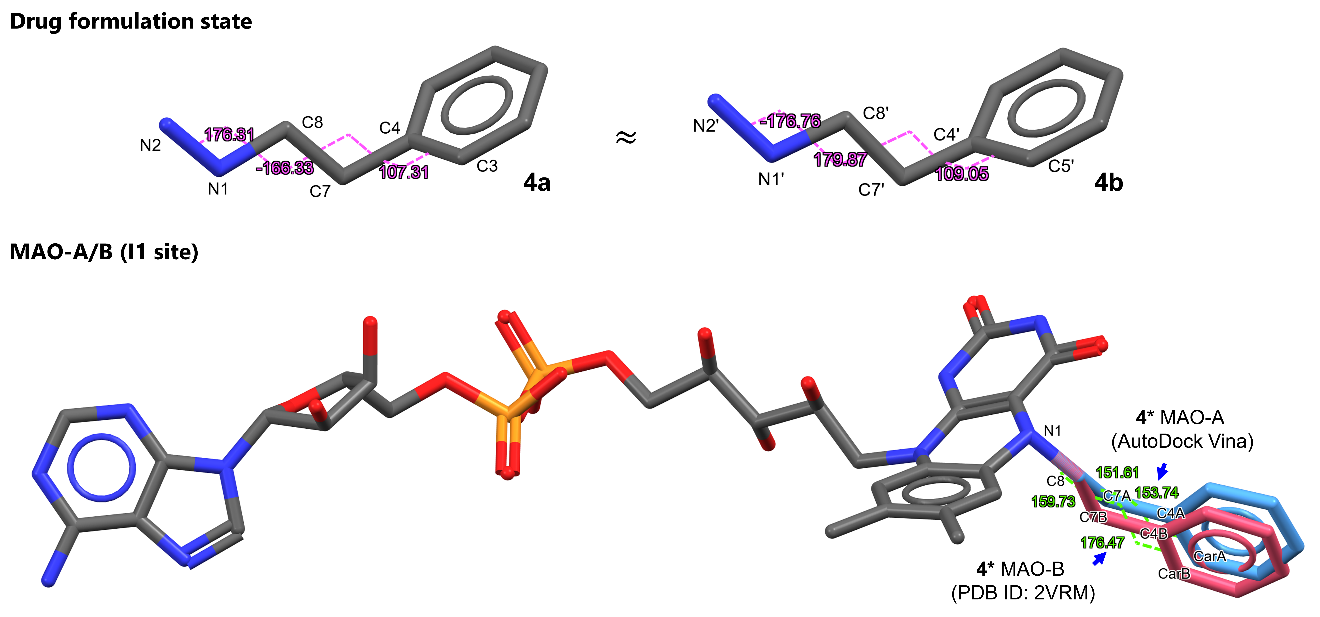


**Supplementary Figure S8** The major conformational changes between the crystal structures of **4** and their docking structures **4*** in MAO-A/B. Phenelzine covalently linked to the FAD in C1 site of MAO-A/B as **4***. Selected torsion angles (C3‒C4‒C7‒C8, C4‒C7‒C8‒N1, C7‒C8‒N1‒N2 in **4a**, C5′‒C4′‒C7′‒C8′, C4′‒C7′‒C8′‒N1′, C7′‒C8′‒N1′‒N2′ in **4b**, C_ar_A‒C4A‒C7A‒C8, C4A‒C7A‒C8‒N1 and C_ar_B‒C4B‒C7B‒C8, C4B‒C7B‒C8‒N1 in **4*** in MAO-A/B) were measured. In the biological state, only the phenylethyl part will covalently bind to the FAD cofactor in MAO-A/B, where the C4‒C7/C4′‒C7′ is presumed to have 44° to 69° rotations. The major torsion changes were highlighted with blue arrows.

**
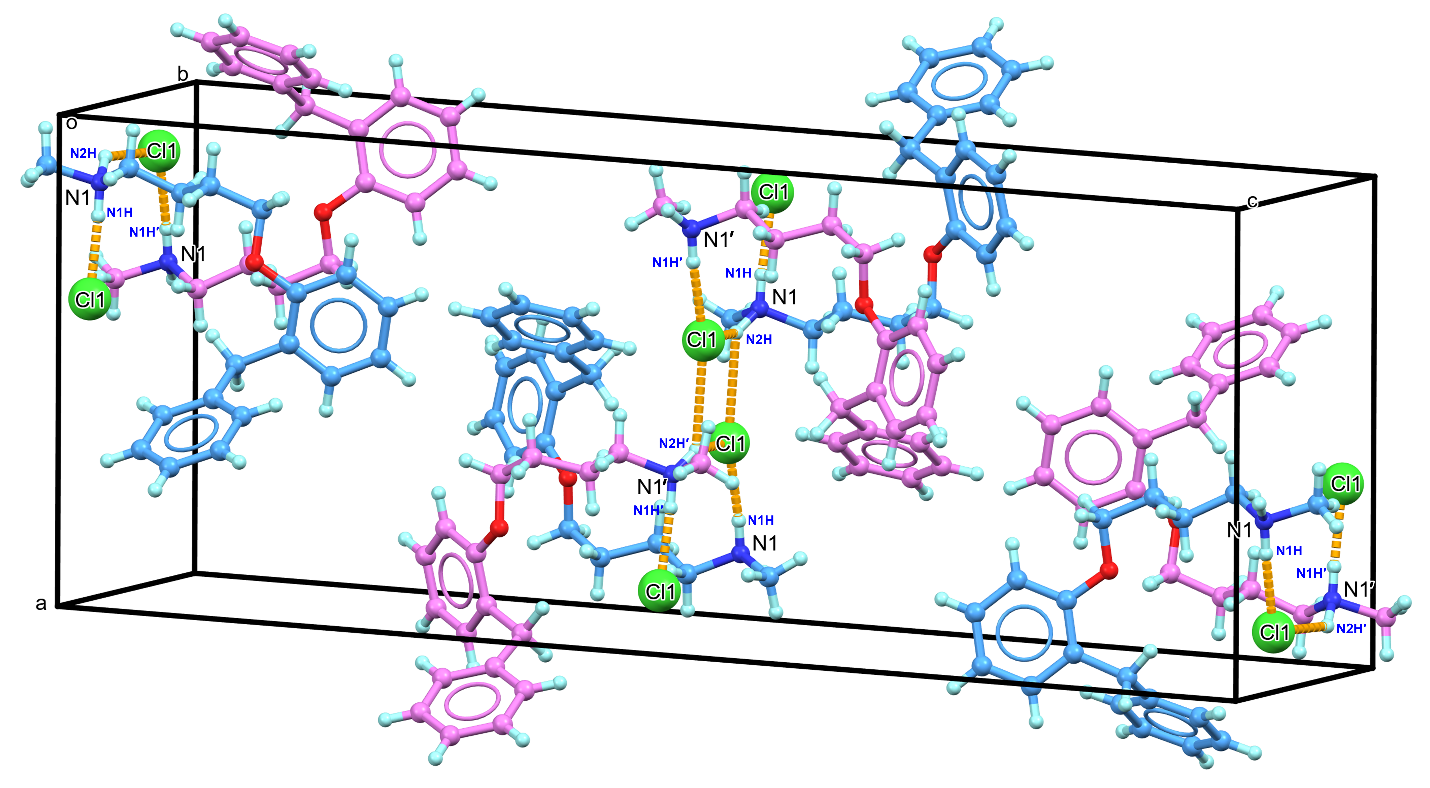
Supplementary Figure S9** The packing diagram of **5**, viewed along the *b*-axis. **5a** was colored in blue, **5b** was colored in violet. Hydrogen bonding interactions were represented by the dashed lines in orange.


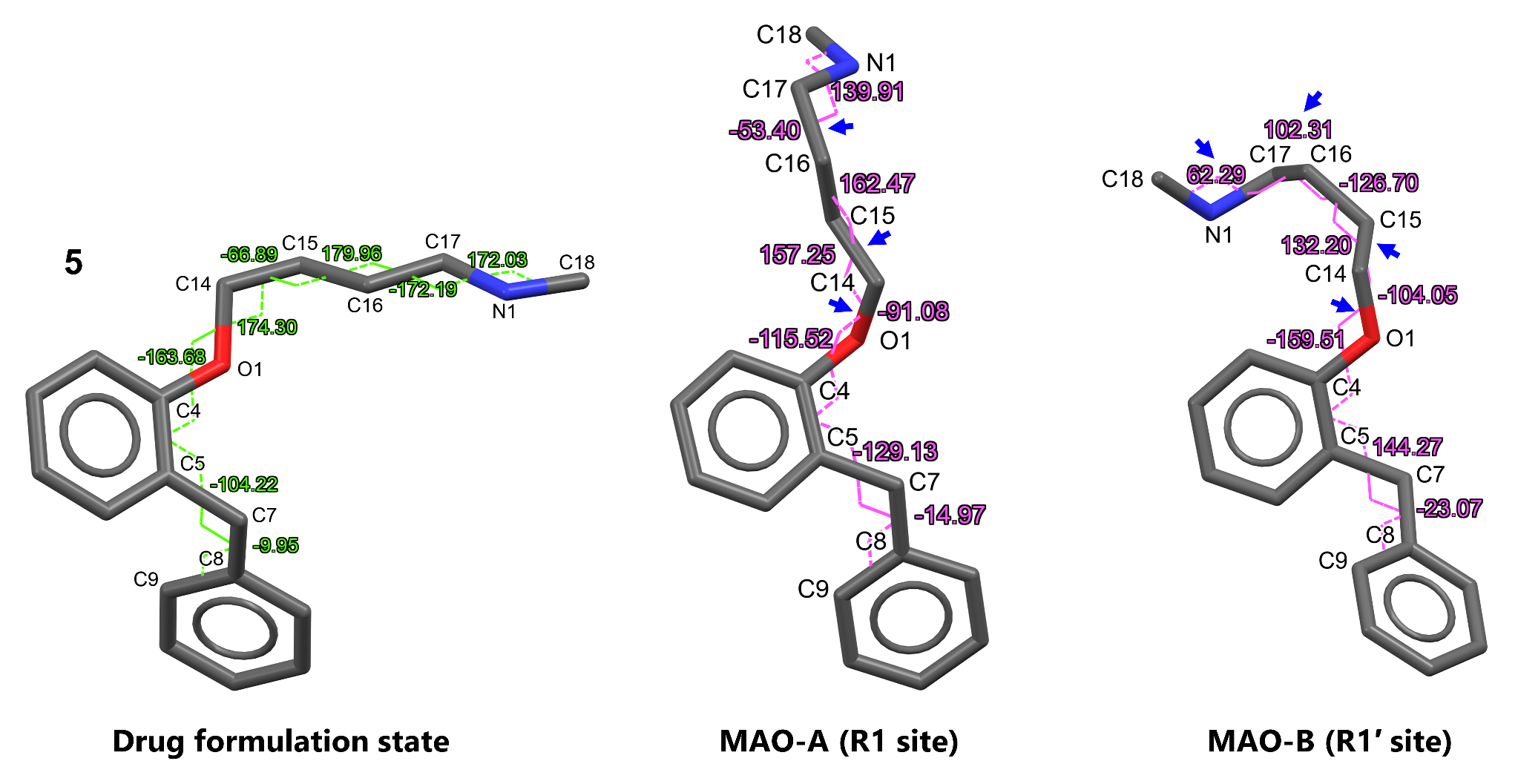


**Supplementary Figure S10** The major conformational changes between the crystal structures of **5** and its molecular docking structures in MAO-A/B. The major torsion changes were highlighted with blue arrows.


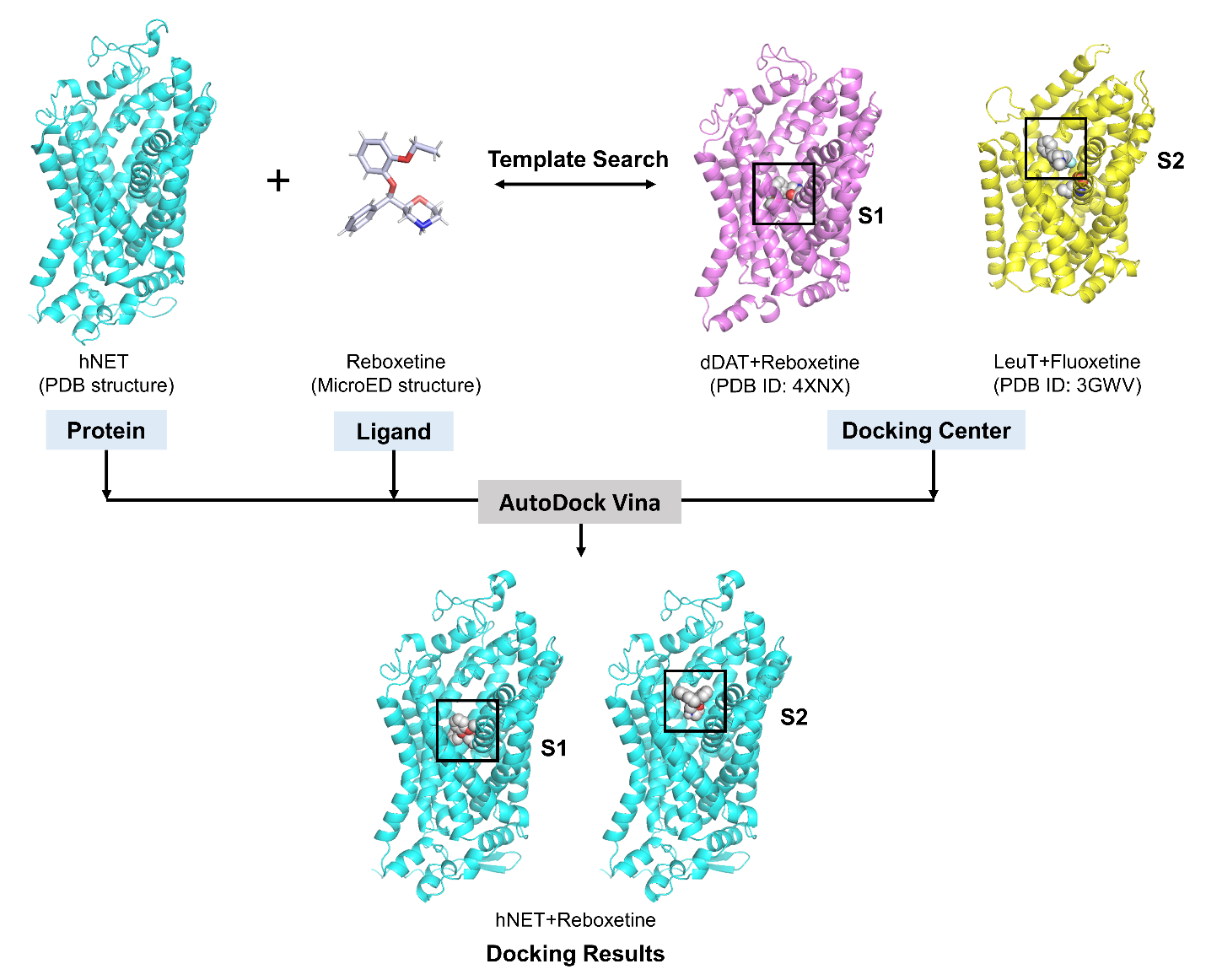


**Supplementary Figure S11** AutoDock Vina docking workflow illustrated by hNET and Reboxetine. The template structures were obtained in PDB database via the query function in CB-Dock2 webtool^13^ or searching the literatures, considering the similarities of both protein and ligand. The docking center was positioned by aligning the protein with template structures, and then used within an 18.75 Å × 18.75 Å × 18.75 Å grid box in AutoDock Vina 1.1.2.^11,12^


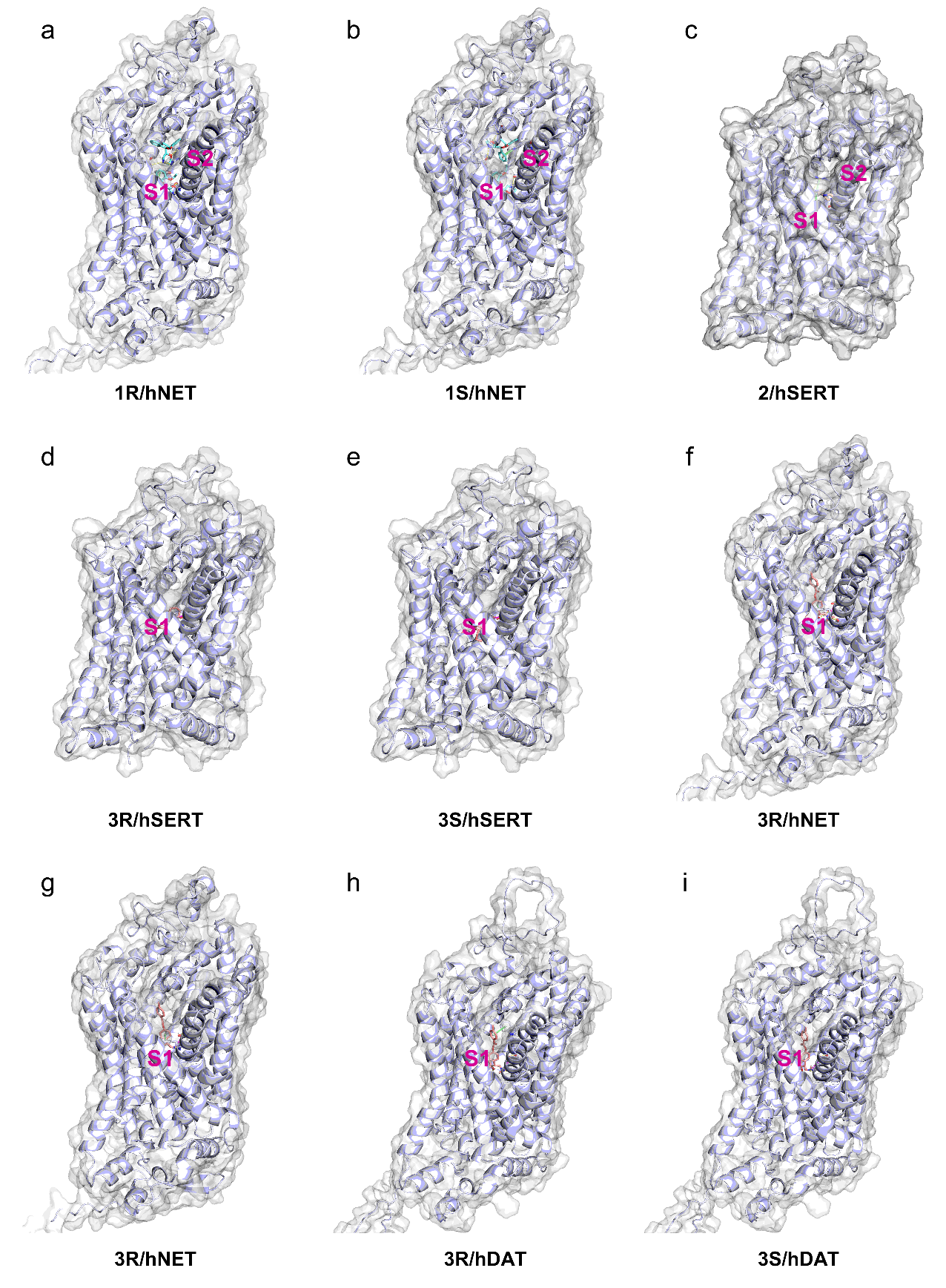


**Supplementary Figure S12** Overall view of protein-drug interaction diagram of complex between (a) **1R** and hNET; (b) **1S** and hNET; (c) **2** and hSERT; (d) **3R** and hSERT; (e) **3S** and hSERT; (f) **3R** and hNET; (g) **3S** and hNET; (h) **3R** and hDAT; (i) **3S** and hDAT. The binding sites were marked.


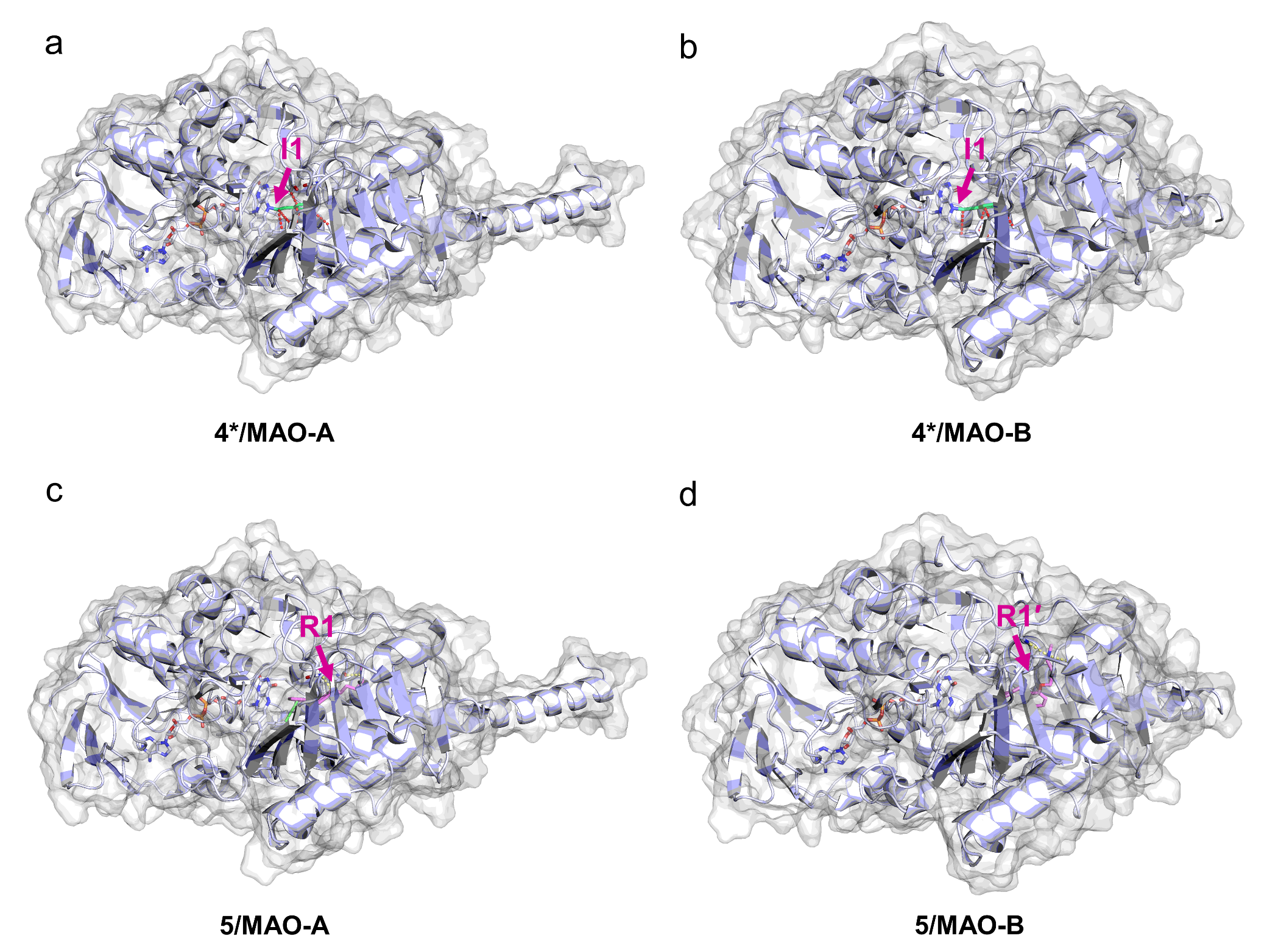


**Supplementary Figure S13** Overall view of protein-drug interaction diagram of complex between (a) **4*** and MAO-A; (b) **4*** and MAO-B; (c) **5** and MAO-A; (d) **5** and MAO-B. The binding sites were marked.


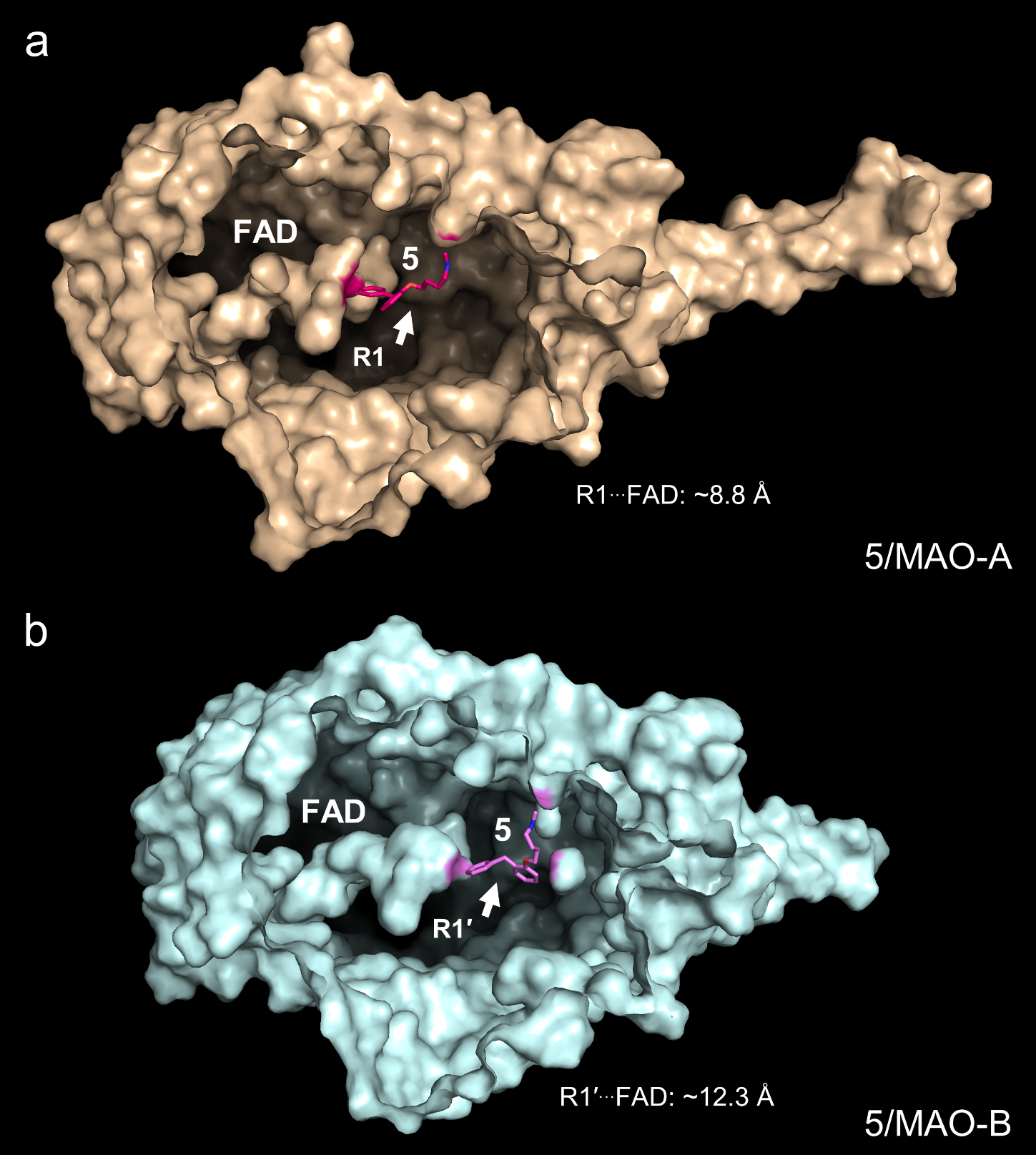


**Supplementary Figure S14** Protein voids observed in **5**/MAO-A and **5**/MAO-B complexes. The distances between FAD and **5** were measured and compared.

**Reference**

1. G. M. Sheldrick, *Acta Crystallogr., Sect. A: Found. Adv.* **2015**, *71*, 3.
2. T. R. Schneider, G. M. Sheldrick, *Acta Crystallogr., Sect. D: Biol. Crystallogr.* **2002**, *58*, 1772.
3. G. M. Sheldrick, *Acta Crystallogr., Sect. C: Struct. Chem.* **2015**, *71*, 3.
4. J. Jumper, R. Evans, A. Pritzel, T. Green, M. Figurnov, O. Ronneberger, K. Tunyasuvunakool, R. Bates, A. Žídek, A. Potapenko, *Nature* **2021**, *596*, 583.
5. A. Penmatsa, K. H. Wang, E. Gouaux, *Nat. Struct. Mol. Biol.* **2015**, *22*, 506.
6. Z. Zhou, J. Zhen, N. K. Karpowich, C. J. Law, M. E. A. Reith, D.-N. Wang, *Nat. Struct. Mol. Biol.* **2009**, *16*, 652.
7. J. A. Coleman, E. M. Green, E. Gouaux, *Nature* **2016**, *532*, 334.
8. H. Wang, A. Goehring, K. H. Wang, A. Penmatsa, R. Ressler, E. Gouaux, *Nature* **2013**, *503*, 141.
9. S.-Y. Son, J. Ma, Y. Kondou, M. Yoshimura, E. Yamashita, T. Tsukihara, *Proc. Natl. Acad. Sci. U.S.A.* **2008**, *105*, 5739.
10. C. Binda, M. Li, F. Hubálek, N. Restelli, D. E. Edmondson, A. Mattevi, *Proc. Natl. Acad. Sci. U.S.A.* **2003**, *100*, 9750.
11. O. Trott, A. J. Olson, *J. Comput. Chem.* **2010**, *31*, 455.
12. J. Eberhardt, D. Santos-Martins, A. F. Tillack, S. Forli, *J. Chem. Inf. Model.* **2021**, *61*, 3891.
13. Y. Liu, X. Yang, J. Gan, S. Chen, Z.-X. Xiao, Y. Cao, *Nucleic Acids Res.* **2022**, *50*, W159.
